# An image cytometric technique is a concise method to detect adenoviruses and host cell proteins and to monitor the infection and cellular responses induced

**DOI:** 10.1186/s12985-017-0888-0

**Published:** 2017-11-10

**Authors:** Takao Morinaga, Thảo Thi Thanh Nguyễn, Boya Zhong, Michiko Hanazono, Masato Shingyoji, Ikuo Sekine, Yuji Tada, Koichiro Tatsumi, Hideaki Shimada, Kenzo Hiroshima, Masatoshi Tagawa

**Affiliations:** 1 Division of Pathology and Cell Therapy, Chiba Cancer Center Research Institute, 666-2 Nitona, Chuo-ku, Chiba, 260-8717 Japan; 2 Department of Molecular Biology and Oncology, Graduate School of Medicine, Chiba University, Chiba, Japan; 3 Division of Respirology, Chiba Cancer Center, Chiba, Japan; 4 Department of Medical Oncology, Faculty of Medicine, University of Tsukuba, Tsukuba, Japan; 5 Department of Respirology, Graduate School of Medicine, Chiba University, Chiba, Japan; 6 Department of Surgery, School of Medicine, Toho University, Tokyo, Japan; 7 Department of Pathology, Tokyo Women’s Medical University Yachiyo Medical Center, Yachiyo, Japan

**Keywords:** Replication-competent adenovirus, Image cytometry, E1A, Hexon, Cleaved caspase-3

## Abstract

**Background:**

Genetically modified adenoviruses (Ad) with preferential replications in tumor cells have been examined for a possible clinical applicability as an anti-cancer agent. A simple method to detect viral and cellular proteins is valuable to monitor the viral infections and to predict the Ad-mediated cytotoxicity.

**Methods:**

We used type 5 Ad in which the expression of *E1A* gene was activated by 5′-regulatory sequences of genes that were augmented in the expression in human tumors. The Ad were further modified to have the fiber-knob region replaced with that derived from type 35 Ad. We infected human mesothelioma cells with the fiber-replaced Ad, and sequentially examined cytotoxic processes together with an expression level of the viral E1A, hexon, and cellular cleaved caspase-3 with image cytometric and Western blot analyses.

**Results:**

The replication-competent Ad produced cytotoxicity on mesothelioma cells. The infected cells expressed E1A and hexon 24 h after the infection and then showed cleavage of caspase-3, all of which were detected with image cytometry and Western blot analysis. Image cytometry furthermore demonstrated that increased Ad doses did not enhance an expression level of E1A and hexon in an individual cell and that caspase-3-cleaved cells were found more frequently in hexon-positive cells than in E1A-positive cells. Image cytometry thus detected these molecular changes in a sensitive manner and at a single cell level. We also showed that an image cytometric technique detected expression changes of other host cell proteins, cyclin-E and phosphorylated histone H3 at a single cell level.

**Conclusions:**

Image cytometry is a concise procedure to detect expression changes of Ad and host cell proteins at a single cell level, and is useful to analyze molecular events after the infection.

**Electronic supplementary material:**

The online version of this article (10.1186/s12985-017-0888-0) contains supplementary material, which is available to authorized users.

## Background

Recombinant replication-competent viruses were demonstrated to produce cytotoxic effects preferentially on tumors and a number of clinical trials were conducted to evaluate efficacy and safety of the viral agents [1]. Herpes simplex viruses expressing *granulocyte macrophage colony stimulating factor* gene, for example, produced significant effects on metastatic melanoma and have been approved in USA and EU [2]. Adenoviruses (Ad) are one of the agents that are relatively easy to be genetically modified and are commonly used to produce replication-restricted types targeting human tumors. Preferential Ad replication in tumors can be achieved by activation of the E1A region with a transcriptional regulatory unit of a gene which is up-regulated in human tumors [3]. Replacing an authentic viral E1A regulatory region with such an exogenous region enable Ad to proliferate in tumors without damaging normal tissues and consequently tumor cell death was induced. We and others previously showed that a 5′ untranslated region of *survivin* (Sur) [4] or *midkine* (MK) [5] gene, which were up-regulated in the expression in a number of human tumors, activated a reporter gene in human tumors but much less in human normal cells. Replication-competent Ad powered by such a regulatory region in fact produced cytotoxicity in various type of human tumors [4, 6]. We also developed Ad in which the fiber-knob region, mediating Ad binding to the cellular receptors [7], was replaced with that of other subtypes. Type 5 Ad use coxsachie adenovirus receptor (CAR) as the main cellular receptor and integrin αvβ3 and αvβ5 as the ancillary receptor, whereas type 35 Ad vector use CD46 as the main receptor [8]. Type 5 Ad bearing the Ad35-derived fiber-knob structure (AdF35) therefore infected CD46-positive cells irrespective of CAR expression [9, 10]. An expression level of CAR molecules in human tumors is often down-regulated, whereas that of CD46 molecules was rather up-regulated in a number of human tumors [11]. AdF35 consequently infected human tumors better than Ad5 [12] and produced greater cytotoxicity [13].

A mechanism of cell death induced by Ad replications can be different from that by conventional chemotherapy. Replication-competent Ad were thereby examined for a possible combinatory use with the anti-cancer agents and recently with immunotherapy [14]. Prediction of Ad-mediated cytotoxicity will be important in a future clinical application but such a predictive biomarker remains unknown in a preclinical study. One of the reasons is a complexity of Ad-mediated cell death since viral replications and subsequent viral spreading are influenced by cellular factors which may affect infection efficacy in an interaction between tumor cells and the microenvironment, anti-viral immune responses and susceptibility of tumors to cell death [15, 16].

Detailed analyses of viral and cellular proteins expressed are crucial for investigating viral replications and induction of cytotoxicity in target cells. Western blot analysis can show expression levels of viral and cellular proteins in a population but is not be suitable for detecting those in a small cell population. In this study, we tested a possible use of image cytometry by detecting the viral early and late proteins together with cellular proteins. Image cytometry can analyze gene expression and the levels of multiple proteins with easy. The present study demonstrated that an image cytometric technique was a handy method to monitor expression of viral and host cell proteins at a single cell level.

## Methods

### Cells

Human mesothelioma, NCI-H2452 and MSTO-211H cells, and a packaging cell line for Ad production, HEK293 cells, were purchased from ATCC (Manassas, VA, USA) and were cultured with RPMI 1640 supplemented with 10% fetal calf serum and penicillin/streptomycin (P4333, Merck, St. Louis, MO, USA) under 5% CO_2_ in air at 37 °C.

### Construction of Ad

AdF35 DNA were produced with the Adeno-X vector (type 5 Ad, Takara, Shiga, Japan) in which the fiber-knob region (accession number: M73260 at 31042-32787) was replaced with that of Ad35 DNA (Avior Therapeutic, Seattle, WA, USA) [17]. Replication-competent AdF35 in which the *E1* gene was activated by an exogenous regulatory element of survivin (AdF35/Sur) or midkine (AdF35/MK) were prepared by replacing the authentic E1 promoter region with 5′-upstream regulatory sequences of the *Sur* (0.5 kb, U75285) [4] or the *MK* (0.6 kb, D10604) [5] gene. Replication-incompetent AdF35 expressing the *β-galactosidase* gene (NM066611) activated by cytomegalovirus promoter (BK000394) (AdF35/LacZ) were prepared with the above Adeno-X vector which were partly replaced with Ad35 DNA [17]. The above Ad DNA was transfected into HEK293 cells and Ad produced were purified with an Adeno-X virus purification kit (Takara) (Additional file 1). The numbers of virus particles (vp) per ml was estimated with the formula, absorbance at 260 nm of purified Ad in the presence of 0.1% sodium dodecyl sulfate × 1.1 × 10^12^ [18].

### Ad infection

Cells (1.6 × 10^5^ cells/ml, 3 × 10^4^ cells/cm^2^) were seeded on tissue-culture grade dishes (353,002, Corning, NY, USA) a day before infection. Ad stock solutions (AdF35/Sur; 1.2 × 10^12^ vp/ml, AdF35/MK; 2.5 × 10^12^ vp/ml, Ad35/LacZ; 1.2 × 10^12^ vp/ml) were diluted with 4 ml of RPMI1640 with 10% fetal calf serum to final virus concentrations depending on respective experiments. Culture medium was then completely replaced with the Ad-containing medium and cells were further cultured for the time indicated. For cytotoxicity test with a dye exclusion assay, cells (2.0 × 10^4^ cells/ml, 4.2 × 10^3^ cells/cm^2^) were seeded on 6-well cell culture plate (353,046, Corning) a day before infection. Ad stock solutions were diluted with 2 ml of RPMI1640 with 10% fetal calf serum to final virus concentrations (10^4^ vp/cell). Infection was performed as above.

### Cytotoxicity of Ad

Infection of cells with Ad was performed by adding Ad onto cells in cell culture and numbers of cells infected with AdF35/Sur were counted with the trypan blue dye exclusion assay [19]. The adherent and floating cells were also counted.

### Immunofluorescence

Cells infected with AdF35/Sur were sequentially treated with 2% paraformaldehyde for 15 min, 0.2% Triton X-100 for 5 min, and 0.1% saponin/3% bovine serum albumin for 30 min. The cells were incubated with anti-Ad type 2/5 E1A antibody (Ab) (sc-25, 1: 50 dilution, Santa Cruz Biotech, Dallas, TX, USA) followed by anti-mouse IgG labeled with Alexa Fluor 488 (A-21202, 1: 500, Thermo Fisher Scientific, Fremont, CA, USA) and RNase A (200 μg /ml) for 1 h. They were further treated with propidium iodide (50 μg/ml) for 30 min for nuclear visualization with a confocal laser microscope, Leica TCS SP8 with a 40× Oil-immersion objective lens (Leica microsystems, Deerfield, Illinois, USA).

### Image cytometry

Cells infected with AdF35/Sur were sequentially treated with 2% paraformaldehyde for 15 min, 0.2% Triton X-100 for 5 min, and 0.1% saponin/3% bovine serum albumin for 30 min. The cells were then incubated with Ab against Ad type 2/5 E1A (1: 50) or Ad hexon E11 (sc-52,365, 1: 50, Santa Cruz Biotech), and with cleaved caspase-3 (#9664S, 1: 200, Cell Signaling, Danvers, MA, USA) for 1 h, followed by anti-mouse IgG labeled with Alexa Fluor 488 (1: 500) or anti-rabbit IgG labeled with Alexa Fluor 555 (ab150074, 1: 500, Abcam, Cambrige, UK) for 1 h. They were analyzed with Tali image-based cytometer (Thermo Fisher Scientific, Fremont, CA, USA).

### Western blot analysis

Cells were lysed with a buffer containing 2% sodium dodecyl sulfate with protease inhibitor cocktail (P8340, Merck), phosphatase inhibitor cocktail solution I (167-24,381, Wako, Osaka, Japan), 0.5 mM EDTA and 5% 2-mercaptoethanol, and were sonicated. Lysate of MSTO-211H cells infected with AdF35/Sur was subjected to sodium dodecyl sulfate polyacrylamide gel electrophoresis [20]. The protein was transferred to a nylon filter and was hybridized with Ab against Ad type 2/5 E1A (1: 200), hexon (1: 200), pRb (#9309, 1: 1000), cleaved caspase-3 (Cell Signaling, 1: 1000), γ-H2A histone family member X (γ-H2AX) (#613401, BioLegend, San Diego, CA, USA, 1: 500), NBS-1 (ab23996, Abcam, 1: 1000), p53 (Ab-6, Clone DO-10, 1: 1600), or tubulin-α (DMIA, Thermo Fisher Scientific, 1:3000) as a loading control. The membranes were developed with the ECL system (GE Healthcare, Buckinghamshire, UK).

## Results

### AdF35/Sur-mediated cytotoxicity

**Fig. 1:**
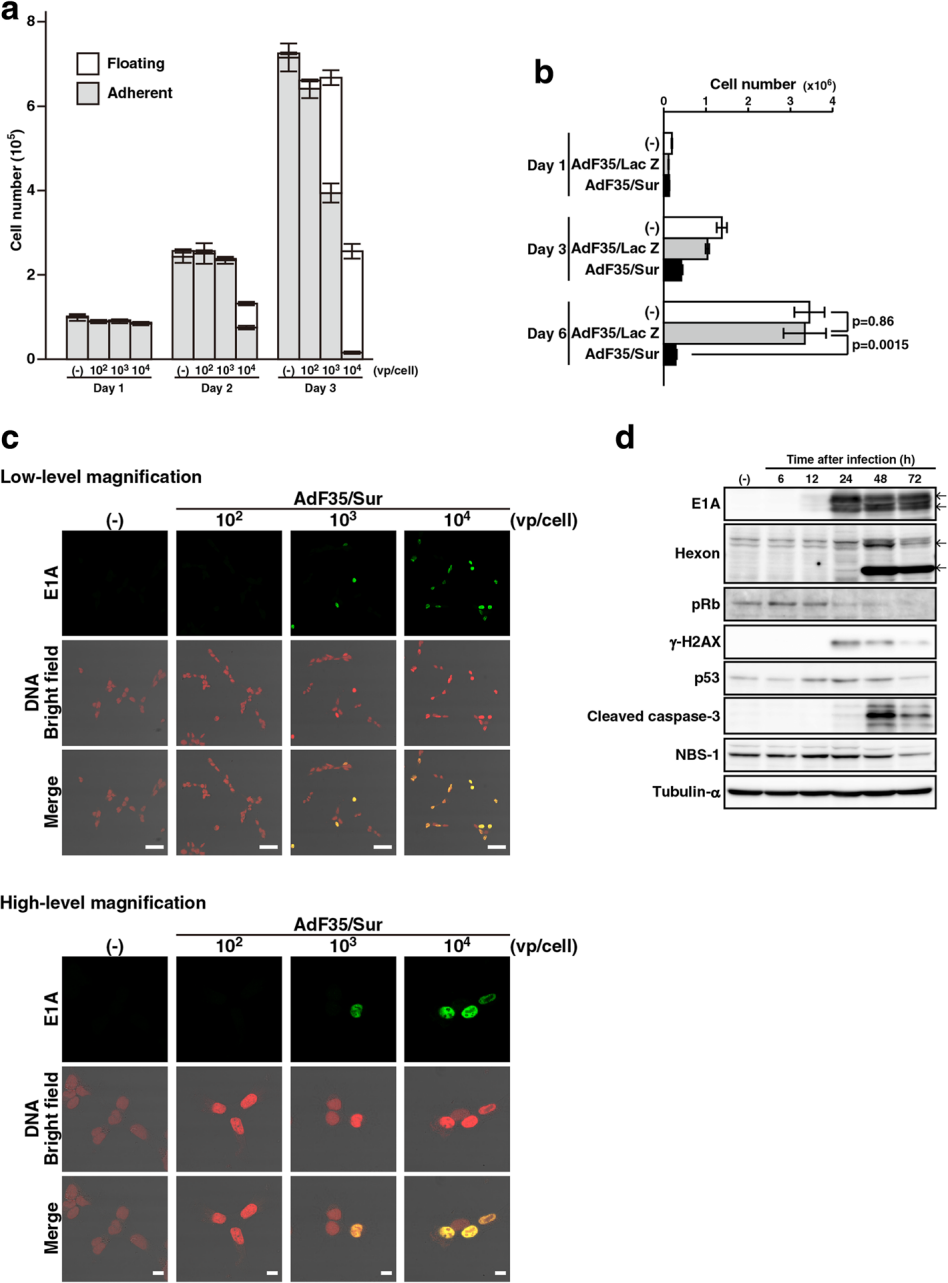
AdF35/Sur-mediated cytotoxicity. **a** MSTO-211H cells were uninfected or infected with AdF35/Sur at a vp/cell dose as indicated, and the numbers of adherent and floating cells were counted. Average and SE bars are shown. (−): uninfected. **b** MSTO-211H cells were uninfected or infected with AdF35/LacZ or AdF35/Sur (10^4^ vp/cell) and live cell numbers were counted with a trypan blue dye exclusion assay. **c** MSTO-211H cells were uninfected or infected with AdF35/Sur at a vp/cell as indicated for 1 day, and were stained with anti-E1A Ab and propidium iodide. **d** MSTO-211H cells were uninfected or infected with AdF35/Sur (10^4^ vp/cell) as indicated and were examined for the expression levels of viral and cellular proteins with Western blot analysis. E1A and hexon showed multiple bands. Tubulin-α was used as a loading control

We examined cytotoxic effects of AdF35/Sur on human mesothelioma, MSTO-211H cells with the wild-type *p53* genotype (Fig. 1). Cells infected with AdF35/Sur became detached from a culture plate and floated in a dose-dependent manner (Fig. 1a). Growth of AdF35/Sur-infected cells were retarded although they were not dead until day 3 (Fig. 1b). The cell numbers then decreased on day 6, indicating that cell death was induced. In contrast, proliferation of AdF35/LacZ-infected cells were minimally suppressed.

An immunofluorescent staining showed that Ad early protein, E1A, was expressed in the nuclei on day 1 (Fig. 1c). We detected expression of E1A from 24 h and that of hexon, one of the Ad late genes, from 48 h after the infection with Western blot analysis (Fig. 1d). We also detected cleavage of caspase-3 from 48 h. These data indicated sequential expression of Ad early gene and late gene products followed by an apoptotic process. AdF35/Sur infection also induced γ-H2AX expression, a DNA damage marker, at 24 h, and expression of NBS-1, a marker for cellular DNA damage responses, was down-regulated upon Ad infection as reported [21]. Furthermore, p53 expression increased due to Ad-induced DNA damages, and pRb expression decreased thereafter.

### Detection of E1A and cleaved caspase-3 with image cytometry

**Fig. 2:**
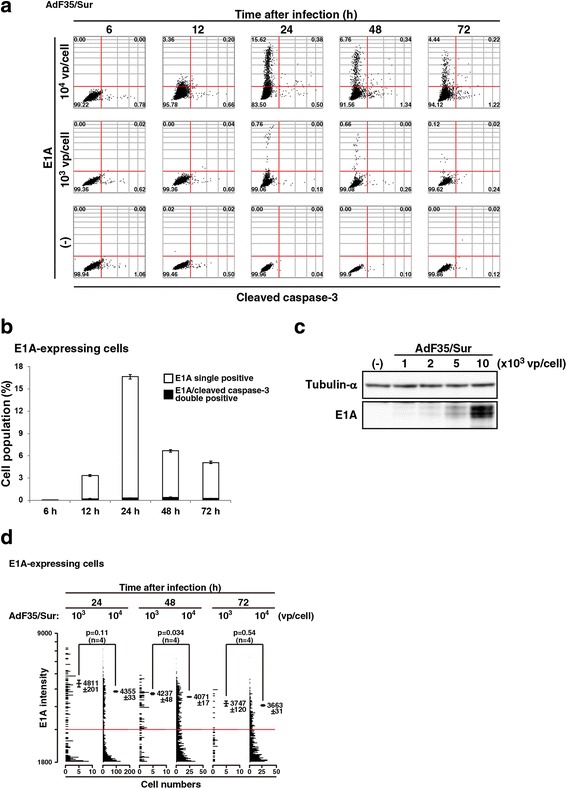
Image cytometric analysis of E1A and cleaved caspase-3. **a** MSTO-211H cells were uninfected or infected with AdF35/Sur (10^3^ or 10^4^ vp/cell) for a period as indicated, stained with Ab against E1A and cleaved caspase-3, and analyzed with Tali image-based cytometer. Representative data are shown. The red lines are tentatively drawn to distinguish stained and unstained cells. A number in image data shows a percentage of the corresponding fraction. **b** Quantitative analysis of E1A single positive and E1A/cleaved caspase-3 double positive cells (10^4^ vp/cell). Averages and SE bars are shown. **c** Western blot analysis to detect E1A expression in MSTO-211H cells uninfected or infected with AdF35/Sur at a vp/cell dose as indicated for 24 h. (−): uninfected. **d** E1A intensity per cell analyzed with image cytometry. The E1A expression per cell, corresponding each dot in (**a**), was plotted and the dot numbers are shown in the abscissa axis. Cells showing less than 1800 (arbitrary unit) at the E1A fluorescent intensity were gated out. A red line shows E1A intensity at 2000 and the E1A intensity of positively stained cells (>2000 units) was calculated. An average unit of the intensity of the positive cells and SE are shown with statistical analysis (ANOVA)

We examined a possible use of image cytometry to detect E1A and cleaved caspase-3 at a single cell level (Fig. 2). A dual staining with anti-E1A and anti-cleaved caspase-3 Ab showed that E1A expression was detected as early as 12 h after the infection, significantly augmented at 24 h and decreased thereafter (Fig. 2a & b). In contrast, cleaved caspase-3 was detected even in untreated cells and the cleavage in infected cells increased after 48 h. Double positive cells for E1A and cleaved caspase-3 were minimally detected. We also examined E1A expression at 24 h with Western blot analysis under the same experimental condition as used in the image cytometric analysis (Fig. 2c). The E1A expression increased in an Ad dose-dependent manner but was scarcely detected in cells infected at 10^3^ vp/cell in contrast to image cytometry which detected E1A-positive cells infected at the same dose. These data collectively indicated that image cytometry enabled us to detect E1A with increased sensitivity. We also calculated an average E1A intensity of the positively stained cells which were infected at 10^3^ or 10^4^ vp/cell (Fig. 2d). The intensity of cells infected at 10^3^ vp/cell was different only at 48 h from that of those at 10^4^ vp/cell although the positive cell numbers were greater in infection at 10^4^ vp/cell than in that at10^3^ vp/cell. A range of the E1A expression level per cell was thus similar irrespective of Ad doses. Increase of E1A expression according to Ad doses, detected with Western blot analysis, was therefore attributable to increased numbers of infected cells but not to an enhanced E1A expression level per cell.

### Detection of hexon and cleaved caspase-3 with image cytometry

**Fig. 3:**
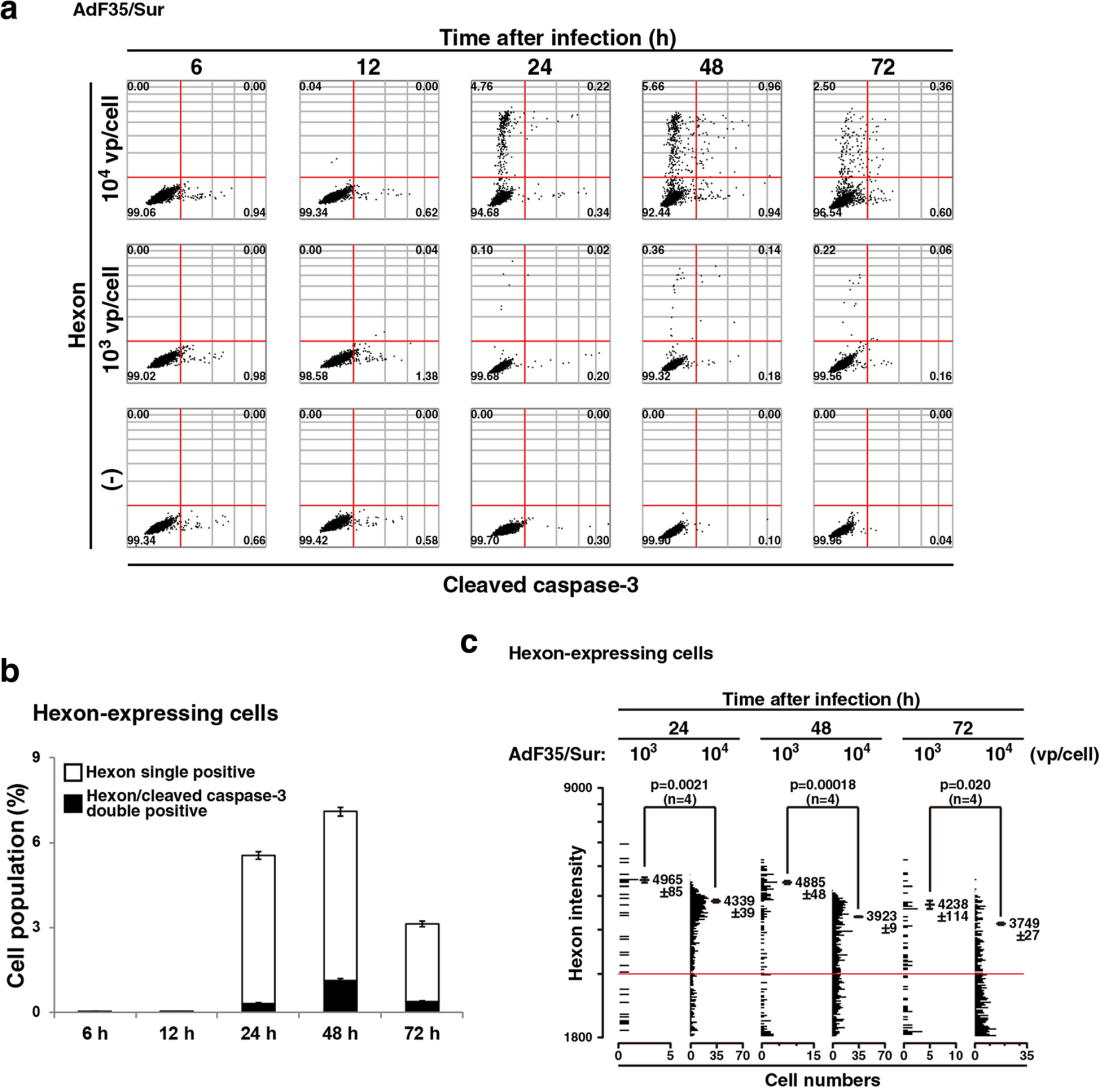
Image cytometric analysis of hexon and cleaved caspase-3. **a** MSTO-211H cells were uninfected or infected with AdF35/Sur (10^3^ or 10^4^ vp/cell) for a period as indicated, stained with Ab against hexon and cleaved caspase-3, and analyzed with Tali image-based cytometer. Representative data are shown. The red lines are tentatively drawn to distinguish stained and unstained cells. A number in image data shows a percentage of the corresponding fraction. **b** Quantitative analysis of hexon single positive and hexon/cleaved caspase-3 double positive cells (10^4^ vp/cell). Averages and SE bars are shown. **c** Hexon intensity per cell analyzed with image cytometry. The hexon expression level per cell, corresponding each dot in (**a**), was plotted and the cell numbers are shown in the abscissa axis. Cells showing less than 1800 (arbitrary unit) at the hexon fluorescent intensity were gated out. A red line shows hexon intensity at 2000 and the hexon intensity of positively stained cells (>2000 units) was calculated. An average unit of the intensity of the positive cells and SE are shown with statistical analysis (ANOVA)

We then stained AdF35/Sur-infected cells with Ab against hexon and cleaved caspase-3 (Fig. 3a). Expression of hexon was detected after 24 h and reached to the maximum at 48 h (Fig. 3b). Cleaved caspase-3 expression was not different from the expression of cells stained dually with anti-E1A Ab (Fig. 2a). Percentages of the cell population positive for both hexon and cleaved caspase-3 were greater than those of double positive cells for E1A and cleaved caspase-3, indicating that caspase-3 was cleaved in a later phase of Ad infection. Interestingly, an expression level of hexon per cell was greater in infection at 10^3^ vp/cell than in infection at 10^4^ vp/cell (Fig. 3c). The hexon level was thus not correlated with virus dose used and these data indicated that infection with a high dose of Ad increased numbers of infected cells but did not augment an expression level of the viral genes per cell.

### Feasibility of image cytometric detection

**Fig. 4:**
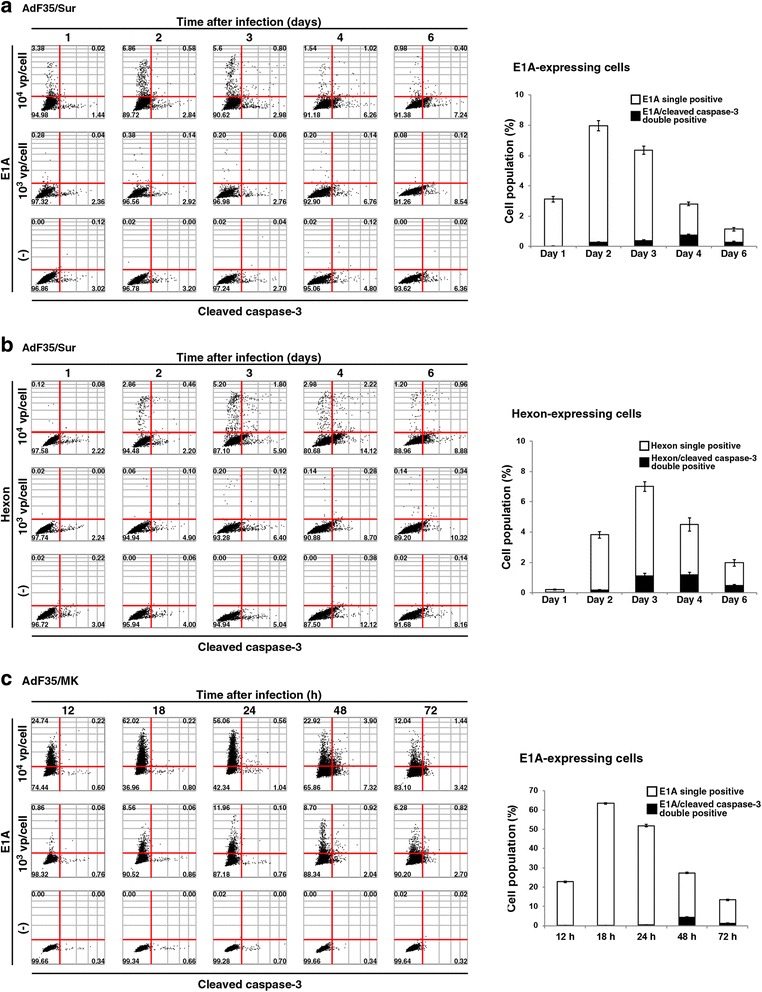
Image cytometric analysis of viral and cellular proteins. **a** NCI-H2452 cells were uninfected or infected with AdF35/Sur (10^3^ or 10^4^ vp/cell) for a period as indicated, stained with Ab against E1A and cleaved caspase-3, and analyzed with Tali image-based cytometer. Representative data are shown. The red lines are tentatively drawn to distinguish stained and unstained cells. A number in image data shows a percentage of the corresponding fraction. (−): uninfected. Bar graphs shows quantitative analysis of E1A single positive and E1A/cleaved caspase-3 double positive cells in (**a**) (10^4^ vp/cell). Averages and SE bars are shown. **b** NCI-H2452 cells were infected as shown in (**a**) and stained with Ab against hexon and cleaved caspase-3. Bar graph shows quantitative analysis of hexon single positive and hexon/cleaved caspase-3 double positive cells in (**b**) (10^4^ vp/cell). **c** MSTO-211H cells were uninfected or infected with AdF35/MK (10^3^ or 10^4^ vp/cell) for a period as indicated, stained with Ab against E1A and cleaved caspase-3, and analyzed with Tali image-based cytometer. Bar graph shows quantitative analysis of E1A single positive and E1A/cleaved caspase-3 double positive cells in (**c**) (10^4^ vp/cell)

We further examined feasibility of image cytometric detection with different cells and replication-competent Ad (Fig. 4). Human mesothelioma NCI-H2452 cells were infected with AdF35/Sur and examined for expression of E1A and hexon together with cleaved caspase-3 (Fig. 4a & b). NCI-H2452 cells had a truncated p53 protein [22] and were relatively resistant to Ad-mediated cytotoxicity. We then examined the expression levels until day 6. Expression of E1A reached to the maximum level on day 2 and decreased thereafter (Fig. 4a). Hexon expression became the highest on day 3 and the level declined thereafter. A dual staining with cleaved caspase-3 demonstrated that cells positive for both E1A and cleaved caspase-3 was few than those positive for hexon and cleaved caspase-3. These data indicated that AdF35/Sur-induced expression profiles of viral and cellular proteins were similar between MSTO-211H cells and NCI-H2452 cells although time to attain the maximum expression was different.

We next tested E1A and cleaved caspase-3 expression levels in MSTO-211H cells infected with a different type of replication-competent Ad, AdF35/MK (Fig. 4c). E1A was detected 12 h after the infection and the expression reached at the maximum at 18 h, whereas cells positive for cleaved caspase-3 became maximum at 48 h in infection at 10^4^ vp/cell. These expression profiles were relatively similar to those of MSTO-211H cells infected with AdF35/Sur although a differential transcriptional activity between the *Sur* and the *MK* gene might influence the viral gene expression.

**Fig. 5:**
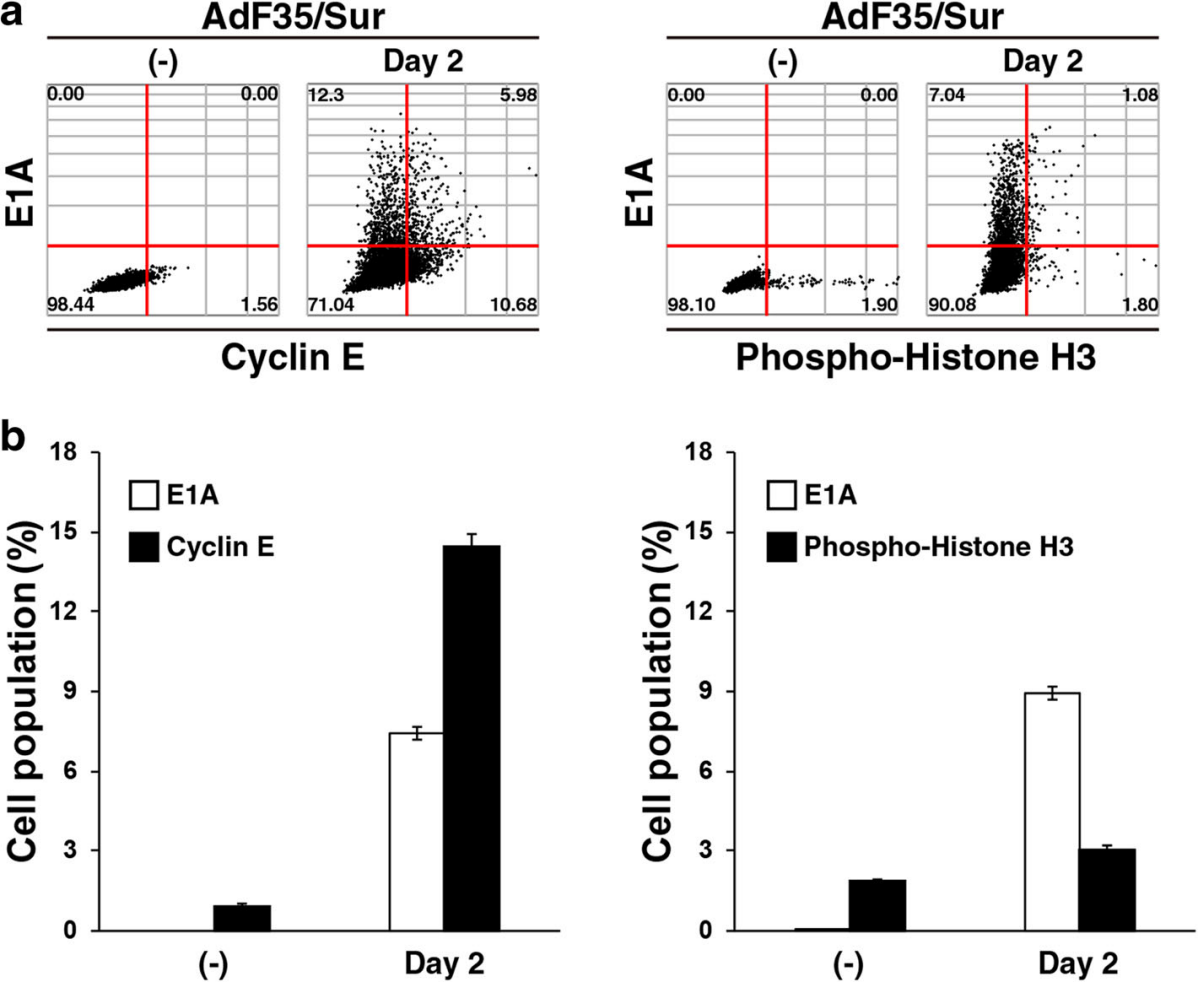
Image cytometric analysis of cellular proteins. **a** MSTO-211H cells were uninfected or infected with AdF35/Sur (10^4^ vp/cell), stained with Ab against cyclin E or phosphorylated histone H3 and E1A, and analyzed with Tali image-based cytometer. Representative data are shown. The red lines are tentatively drawn to distinguish stained and unstained cells. A number in image data shows a percentage of the corresponding fraction. (−): uninfected. **b** Quantitative analysis of E1A-, cyclin E- and phosphorylated histone H3-positive cells in (**a**). Averages and SE bars are shown

We also examined expression of cyclin E and phosphorylated histone H3 in combination with E1A expression with image cytometry (Fig. 5a). Ad infection facilitated S-phase entry and M-phase exit in cell cycle and we used cyclin E and phosphorylated histone H3 as a maker for S-phase and M-phase, respectively [23, 24]. MSTO-211H cells significantly increased percentages of cyclin E-positive cells 2 days after AdF35/Sur infection (uninfected cells; 0.9 + 0.09%, infected cells; 14.4 + 0.52%), whereas those marginally increased percentages of cells positive for phosphorylated histone H3 (uninfected cells; 1.9 + 0.07%, infected cells; 3.0 + 0.18%), (Fig. 5b). These data were consistent with the previous studies [23, 24] and demonstrated that an image cytometric analysis detected viral and cellular proteins at a single cell level.

## Discussion

We investigated a possible image cytometric analysis of Ad early and late proteins together with cellular proteins. An image cytometry technique detected Ad proteins, E1A and hexon molecules, and cellular proteins, cleaved caspase-3, cyclin E and phosphorylated histone H3 molecules, at a single cell level. The technique is concise with less amounts of cells required in comparison with Western blot analysis and enables us to investigate molecular expression profiles quantitatively.

We detected E1A expression in cells infected with replication-competent Ad and compared detection sensitivity between image flow cytometer and Western blot analysis with the same Ab. We may not able to conclude differential sensitivity since a detection level is influenced by many factors including affinity of the second Ab used. Nevertheless, an image cytometry technique could detect a small fraction of Ad-infected population as being E1A-positive in contrast to Western blot analysis that detected the E1A level in a whole cell population infected with a high Ad dose. We can therefore conclude that an image cytometric analysis is more sensitive to detect viral proteins than Western blot analysis. We also analyzed hexon, one of the Ad major proteins, and cleaved caspase-3, cellular protein involved in apoptosis, and demonstrated that E1A expression preceded hexon expression and cleaved caspase-3 was expressed preferentially in hexon-positive cells rather than E1A-positive cells. These data are quite understandable in terms of Ad replication processes. We also noticed that a range of an E1A or a hexon expression level in a single cell was not correlated with Ad doses used. The data indicated that Ad infection with a greater dose increased cell numbers infected but did not enhance the viral gene expression levels in each cell. The levels of viral genes expressed in a single cell were thus relatively constant or limited, which was not revealed by a protein blot analysis. The flow cytometry also has a limited ability to estimate number of infectious viral particles since viral particle numbers and the gene expression levels were not directly correlated.

We also showed that image cytometry detected an expression profile of cyclin E and phosphorylated histone H3 A, and indicated that the technique was widely applicable with different cells and with different types of replication-competent Ad. A dual image cytometric technique therefore is useful to analyze expression profiles at one cell level, which gives much of quantitative information in comparison with an immunoprecipitation-Western blot method.

Detection of viral gene products with flow cytometry was reported back to 1990s [25] and the techniques was also used to identify Ad-infected cells [26]. Ad vector bearing *green fluorescent protein* gene expanded utility of a cytometric analysis to identify the virus-infected cells [27]. Moreover, a previous study compared sensitivity and utility of a cytometric technique with other methods such as a quantitative polymerase chain reaction, and demonstrated an advantage of detecting E1A and hexon with flow cytometry over the other procedures [28]. An application of recombinant Ad for an anti-cancer agent further augmented values of molecular imaging with a fluorescent dye in terms of detection of target cells and estimation of infectivity [29–31]. We showed in the present study possible benefits of an image cytometric analysis to detect viral and cellular gene products for analysis of Ad infectivity and for prediction of the cytotoxicity. A meticulous sample processing technique is required to analyze protein expression in a clinical setting since the tumor specimens are mixture of tumor cells and stroma, and often contain dead cells. Image cytometry can detect respective proteins expressed on target cells with easy by gating out non-tumorous and dead cells with appropriate Ab. Detection of multiple gene products with different color dyes, which are now available, makes it possible to analyze complexity of interaction between viral and host cell proteins.

## Conclusions

Image cytometry is a simple and precise assay method to detect viral and cellular proteins at a single cell level. It requires small cell numbers and is useful for detailed analysis of viral infection processes and cellular responses to them. The technique can also be useful for investigating the expression profiles in a time-dependent manner.

## Additional file

Additional file 1: Detailed information as for Ad construction. (DOCX 43 kb)

## Abbreviations

Ab: Antibody
Ad: Adenoviruses
AdF35: Adenovirus vector bearing the type-35-derived fiber-knob structure
AdF35/LacZ: AdF35 expressing the *β-galactosidase* gene
CAR: Coxsachie adenovirus receptor
MK: Midkine
Sur: Survivin
vp: Virus particles
γ-H2AX: γ-H2A histone family member X
