# Supplementary material for: An image cytometric technique is a concise method to detect adenoviruses and host cell proteins and to monitor the infection and cellular responses induced
Source: Virol J. 2017 Nov 10;14:219. doi: 10.1186/s12985-017-0888-0 (PMC5681831; doi:10.1186/s12985-017-0888-0)
Supplement: Additional file 1: — Detailed information as for Ad construction. (DOCX 43 kb) [file 12985_2017_888_MOESM1_ESM.docx]

**Detailed information as for Ad construction**

(1) We first constructed pAd5F35 which encoded type 5 Ad with type 35-derived fiber knob region as follows. The fiber knob region of type 5 Ad which included a coding region for the CAR-binding site between Eco RI sites (31042-32787, GenBank accession number: M73260) was digested with Eco RI and the fiber knob region was replaced with Eco RI fragment of RHSP Ad35 (type 35 Ad, Avior Therapeutics, Seattle, WA, USA) which included a coding region for the CD46-binding site between Eco RI sites (30956-31798, AY271307).

(2) We next constructed a modified pShuttle2 vector (Takara), pS-PL vector, in which the cytomegalovirus-promoter region was removed from the Mun I-Nhe I site and a multi-cloning site with the recognition sequences for Mun I-Sca I-Bam HI-Eco RV-Sal I-Nhe I-Dra I-Apa I-Xba I-Not I-Bst XI-Kpn I-Aff II was inserted.

(3) We removed the authentic transcriptional regulatory region of *E1* region (341-548, M73260), and placed an exogenous region from survivin or midkine gene into the 5’ region of the *E1* region to control the *E1* transcription under the exogenous region. We produced the DNA which did not contain the exogenous region using the following DNA. Bam HI/Mun I-digested LHSP vector (Avior Therapeutics), Xba I/Mun I-digested (corresponding to 1338 and 3923, M73260) the pXC1 vector (Microbix Biosystems, Tronto Ontario, Canada) and a PCR-made DNA fragment (corresponding to 549-1344, M73260) using the pXC1 vector (Microbix Biosystems) as a template. The PCR fragment was created to have Bam HI and Xba I sites and was ligated the above two DNA with these restriction sites, and the ligated DNA was then inserted into pS-PL vector (pS-PL/E1). The final construction contained DNA corresponding to 22-341 and 549-5784 sequences but defective of 342-548 sequences.

(4) We also created PCR products of 5’-upstream regulatory sequences of the *Sur* (-478/+43, U75285, transcription initiation site; +1) or the *MK* (-559/+50, D10604). We then insert these DNA, exogenous regulatory regions into pS-PL/E1 vector at Eco RV site.

(5) DNA created in step (4) and pAd5F35 were digested with I-Ceu I and PI-Sce I, and were ligated. The ligated DNA were further digested with Swa I to digest unligated pAd5F35.

(6) The constructed Ad DNA was amplified with *E. coli* (DH5α) and then was transfected into HEK293 cells following the manufacturer’s instructions Adeno-X expression system (PT3414-1, Takara). Ad were purified with Adeno-X virus purification kit (PT3680-1, Takara).
